# Overexpression of a Soybean Ariadne-Like Ubiquitin Ligase Gene *GmARI1* Enhances Aluminum Tolerance in Arabidopsis

**DOI:** 10.1371/journal.pone.0111120

**Published:** 2014-11-03

**Authors:** Xiaolian Zhang, Ning Wang, Pei Chen, Mengmeng Gao, Juge Liu, Yufeng Wang, Tuanjie Zhao, Yan Li, Junyi Gai

**Affiliations:** National Key Laboratory of Crop Genetics and Germplasm Enhancement, National Center for Soybean Improvement, Key Laboratory for Biology and Genetic Improvement of Soybean (General, Ministry of Agriculture), Nanjing Agricultural University, Nanjing, Jiangsu, China; Key Laboratory of Horticultural Plant Biology (MOE), China

## Abstract

Ariadne (ARI) subfamily of RBR (Ring Between Ring fingers) proteins have been found as a group of putative E3 ubiquitin ligases containing RING (Really Interesting New Gene) finger domains in fruitfly, mouse, human and Arabidopsis. Recent studies showed several RING-type E3 ubiquitin ligases play important roles in plant response to abiotic stresses, but the function of ARI in plants is largely unknown. In this study, an ariadne-like E3 ubiquitin ligase gene was isolated from soybean, *Glycine max* (L.) Merr., and designated as *GmARI1*. It encodes a predicted protein of 586 amino acids with a RBR supra-domain. Subcellular localization studies using Arabidopsis protoplast cells indicated GmARI protein was located in nucleus. The expression of *GmARI1* in soybean roots was induced as early as 2–4 h after simulated stress treatments such as aluminum, which coincided with the fact of aluminum toxicity firstly and mainly acting on plant roots. In vitro ubiquitination assay showed GmARI1 protein has E3 ligase activity. Overexpression of *GmARI1* significantly enhanced the aluminum tolerance of transgenic Arabidopsis. These findings suggest that *GmARI1* encodes a RBR type E3 ligase, which may play important roles in plant tolerance to aluminum stress.

## Introduction

Ubiquitination is an enzymatic, protein post-translational modification by which proteins are selectively targeted for a variety of cellular processes including DNA transcription and repair, cell cycle and division, response to stresses and many others [1]. This process is carried out by three types of enzyme, including an ubiquitin-activating enzyme (E1), an ubiquitin-conjugating enzyme (E2), and an ubiquitin protein ligase (E3) [2]. Encoded by a large gene family of widely divergent isoforms [3], E3 ligases play important roles in governing the ubiquitin signaling pathway by transferring ubiquitin from E2 conjugation to specific protein substrates. E3 ligases are generally divided into two families, with either a HECT or RING-finger domain(s) [1], [4]. The RING-type E3 ubiquitin ligases are generally identified by the presence of conserved cysteine- and histidine- rich RING finger motifs that coordinate zinc atoms [5]. Recently several RING-type E3 ubiquitin ligases were found to play important roles in plant responses to abiotic and biotic stresses. The pepper E3 ubiquitin ligase RING1 gene, *CaRING1*, is required for cell death and the salicylic acid (SA)-dependent defense response [6]. *AtAIRP1* and *AtAIRP2* play roles in abscisic acid (ABA)-mediated drought stress responses in Arabidopsis [7]. In soybean, a RING-finger protein encoded by *GmRFP1* was identified and shown to be involved in ABA signaling and stress responses through the ubiquitin-proteasome pathway [8].

RBR (Ring Between Ring fingers) proteins are characterized by the presence of their RING1 – IBR – RING2 supra-domain, which is composed of two RING finger domains plus an IBR (In Between Rings) domain [9]. Many RBR proteins are known to have E3 ubiquitin ligase activity [10]. ARIADNE (ARI) proteins, a subclass of RBRs, have been identified in fruitfly [11], mouse [12], [13], human [14]–[16], and Arabidopsis [17], [18]. ARI proteins are characterized by the presence of an N-terminal acid-rich cluster, followed by a C3HC4 RING-finger motif, a central IBR or B-box, a second C3HC4 RING-finger structure, and Leu-rich domain at the C terminus. ARI proteins share their RBR domain with PARKIN, a protein involved in autosomal recessive familial Parkinson's disease [9]. PARKIN functions as E2-dependent ubiquitin-protein ligase [17]. Recent studies suggest that the ARI/PARKIN proteins define a new class of RING-finger E3 ligases [19]. There are only few studies on ARI proteins in plants. Recently *AtARI12* in Arabidopsis was identified to be involved in UV-B signaling pathway [20].

Soybean (*Glycine max* [L.] Merr.) is widely grown as a major source of vegetable oil and protein. Soybean quality and yield are affected by various abiotic and biotic stresses. Soybean is also an important crop grown in South China, where acid soils comprise approximately 21% of the total land area [21], [22]. Aluminum (Al) toxicity is a major limiting factor of plant growth and crop production on acidic soils. There is large variation in Al tolerance among soybean varieties, and most of the Al tolerant varieties are from China [23]. Another study showed many Al tolerant varieties are from South China [24]. However, the genes underlying the Al tolerance in soybean remain largely unknown, except recently a soybean malate transporter gene *GmALMT1* which was shown to mediate root malate efflux which underlies soybean Al tolerance [25].

Increasing evidence indicates that RING-type E3 ubiquitin ligases play important roles in plant response to abiotic stresses. However, to date, there are no reports on the functions of soybean ARI proteins. Therefore, in this study, an ariadne-like E3 ubiquitin ligase gene *GmARI1* was cloned from soybean, and its gene expression patterns in different soybean tissues were studied. The transcriptional changes of *GmARI1* in response to various stress such as aluminum (Al) and plant hormone treatments were investigated using real-time quantitative PCR (qRT-PCR). We further characterized the *GmARI1* gene function by its subcellular location, in vitro ubiquitination assay, and performance of the transgenic Arabidopsis overexpressing *GmARI1* under Al stress. The possible mechanisms and signal pathways involved in soybean response to Al are also discussed.

## Materials and Methods

### Plant materials

Seeds of the soybean (*Glycine max* [L.] Merr.) cultivar Nannong 1138-2, provided by the National Center for Soybean Improvement (Nanjing, China), were germinated in sand under 25°C, 60% relative humidity (RH) and a photoperiod of 16 h/8 h (light/dark) cycle (light intensity was about 110 µmol photons. m^−2^s^−1^). Nannong 1138-2 is a released cultivar adapted to South China, which has good agronomic traits and moderate Al tolerance. The soybean plants at VE stage (emergence) were transferred to the ‘standard’ nutrient solution [26], and grown for another ten days before various stress and hormone treatments. The nutrient solution was renewed every five days.

### Isolation of the *GmARI1* gene from soybean

The full-length opening reading frame of the *GmARI1* gene was obtained by RT-PCR using soybean RNA. Total RNA was isolated using Trizol Reagent (Invitrogen, USA) according to the user's manual. 0.2 µg of the purified total RNA was used to synthesize first-strand cDNA by the MMLV-reverse transcriptase (TaKaRa). The primers: 5′-TCCCAATTCTTCTTCTGCCCTAG-3′ and 5′-GCAACCTTTCTTCCAAG CCTTAC -3′ were designed to amplify the *GmARI1* gene located on Chromosome 11. The PCR products were cloned into the pGEM-T vector (Promega) and sequenced (Invitrogen). The sequencing results showed that two *ARI* genes, *GmARI1* and *GmARI2*, were isolated using above primers, due to their high similarity of 97%.

### Sequence analysis

Protein domains were analyzed by the SMART (Simple Modular Architecture Research Tool) (http://smart.embl-heidelberg.de/) and Pfam (Protein families database of alignments and HMMs). The molecular mass, isoelectric point and secondary structure were predicted using ProtParam and SOPMA on the ExPASY(http://www.expasy.org/tools/). The BLASTP program at GenBank (http://www.ncbi.nlm.nih.gov/blast) was used to search the homologous sequences of GmARI1/GmARI2 from Non-Redundant (NR) database. Alignment was performed with ClustalW2 (http://www.ebi.ac.uk/Tools/msa/clustalw2/) and MUSCLE (http://www.ebi.ac.uk/Tools/msa/muscle/) using the default settings. The phylogenetic tree was constructed by the neighbor-joining algorithm (NJ) using MEGA version 5 with 1000 bootstraps.

### Semi-quantitative RT-PCR

To study the tissue expression pattern of *GmARI1*, soybean roots, stems, leaves, and shoot apical meristem (SAM) were collected from 15-day-old plants, flowers and pods were collected from plants at 20 days after flowering (DAF). All tissues were frozen immediately in liquid nitrogen and stored at −80°C. The semi-quantitative RT-PCR assay was performed with 0.1 µg RNA as template for cDNA synthesis. Primers 5′-CTCCATTCTCCATTCTCCTCCTTTGC-3′ and 5′-GTCGTCGTCGCTGTAGTAGT CC -3′ were used for *GmARI1*. Primers:5′-ATCTCATTCCCTTCCCTCGTCTG-3′ and 5′-CTGCCTCTGTGAACTCCATCTCG -3′ were used for *Tubulin-3* (GeneBank accession No. U12286) as the internal control. The PCR products were examined by electrophoresis in 2.0% agarose gel.

### Transient expression of the GmARI1-GFP fusion protein

*GmARI1-GFP* was cloned into pMDC83 vector, with the expression driven by the cauliflower mosaic virus 35S promoter. The ORF of *GmARI1* was amplified by PCR using primers:5′-GGGGACAAGTTTGTACAAAAAA GCAGGCTTCCCAATTCTTCTTCTGCCCTAG-3′ and 5′-GGGGACCACTTTGTACA AGAAAGCTGGGTCTCGACGTTGTTGATAGCACATCTG -3′, without the stop codon. The *35S*-*GmARI1-GFP* in-frame fusion construct and control vector of *35S*-*GFP* were introduced into the Arabidopsis protoplasts cells by PEG-mediated protocol [27], [28]. After culturing in dark at 23°C, the localization of GFP was observed with a confocal microscope the next day (Leica TCS SP2).

### Stress treatments

The plants were put in the ‘standard’ nutrient solution [26] with one of the following chemicals for various treatments: Al stress (10 µM Al(NO_3_)_3_, pH 4.3), drought (osmotic) stress (400 mM mannitol), salt stress (200 mM NaCl), absicisic acid (ABA,100 µM), indoleacetic acid (IAA, 100 µM), jasmonic acid (JA, 100 µM), and salicylic acid (SA, 150 µM), with ‘standard’ nutrient solution (Mg^2+^ was withdrew for Al treatment) as control. The leaves and roots were harvested at 0, 0.5, 1, 2, 4, 6, 8, 12, 24, and 48 h after each treatment. Each sample was the mixture of three seedlings and each treatment was repeated three times. All samples were immediately frozen in liquid nitrogen and stored at −80°C for later use.

### Real-time quantitative PCR

RNAs of different treatments were extracted using Trizol reagent (Invitrogen) and purified with RNase-free DNase I. The cDNA was synthesized from 0.2 µg RNA in a 10 µl reaction volume using PrimeScript R 1st Strand cDNA Synthesis kit (TaKaRa). Primers for *Tubulin-3* (GeneBank accession No. U12286) were 5′- TCATTCCCTTCCCTCGTCTGC-3′ and 5′-CCTCCTTGGTGCTCATCTTGC-3′. Primers for *GmARI1* were 5′-CGCTGGTTCCTGAATTTCCCTTG-3′ and 5′- GTCGTCGTCGCTGTAGTAGTCC-3′. Quantitative real-time PCR was performed with SYBR Green method on ABI 7500 Fast Real-Time PCP system. The following procedure was used for qPCR: 95°C for 5 min; 40 cycles of 95°C for 3 sec; 60°C for 30 sec; 72°C for 30 sec. Data was analyzed using the 2^−ΔΔCT^ method as described by Livak and Schmittgen [29].

### In vitro E3 ubiquitin ligase activity assay of GmARI1 protein

The full-length cDNA of GmARI1 with SalI/XhoI restriction enzyme sites was amplified by PCR, using primers ARI1-F: 5′- ACGCGTCGACATGGAGTCAGAGGATATGCAC-3′ and ARI1-R: 5′-CCGCTCGAGTCGACGTTGTTGATAGCACATCTG -3′. The fragment was cloned into the vector pET28a via the SalI/XhoI restriction sites, with 6×His tag fused to GmARI1 at the N-terminal. The expression construct (pET28a-His_6_-GmARI1) was transformed into *E.coli* BL21 (DE3) cells to produce a recombinant His_6_-GmARI1 fusion protein with an expected mass of about 66 kDa. The transformed cells harboring pET28a-His_6_-GmARI1 were grown at 37°C with vigorous shaking until an OD600 of 0.4–0.6 is reached and induced with 0.1 mM isopropylthio-b-galactoside (IPTG) for 12 h at 16°C. The overexpressed His_6_-GmARI1 was purified using Ni–NTA resin according to the supplier's instructions (GE life sciences). The protein concentration was determined as described by Bradford [30] using BSA as a standard.

For the autoubiquitination assay, each reaction (30 µl final volume) contained 10 µg of recombinant ubiquitin (Ub, Sigma), 0.1 µg rabbit E1 (Boston Biochemicals), 0.2 µg human E2 (UbcH5b, Boston Biochemicals), 2 mM ATP, 50 mM Tris–HCl (pH 7.5), 5 mM MgCl2, and 2 mM DTT contained 500 ng purified His6-GmARI1 [31]. After incubation at 30°C for 2–3 h, the reactions were stopped with sodium dodecyl sulfate-polyacrylamide gel electrophoresis (SDS-PAGE) loading buffer at 95°C for 5 min. The reaction samples were electrophoretically separated on 12% SDS-PAGE gels and transferred on two PVDF membranes separately. The two membranes were blocked and thereafter blotted with an anti-ubiquitin monoclonal antibody (Santa Cruz Biotechnology) and an anti-His_6_ monoclonal antibody (Sigma, USA) for 6 h at a 1∶3000 dilution, respectively. After extensive washing, each of the bound primary antibody was detected with a horseradish peroxidase-conjugated goat anti-rabit IgG secondary antibody using the 3,3′-diaminobenzidine (DAB) development kit according to the manufacturer's protocol (Bio Basic Inc, Canada).

### Generation of *GmARI1* transgenic Arabidopsis

The *GmARI1* gene was amplified by RT-PCR as described above and cloned into the plant expression vector pMDC83 under the control of CaMV 35S promoter by Gateway technology (Invitrogen), and the recombined plasmid was transferred into *A. tumefaciens* strain EHA105. Arabidopsis plants (Col-0 ecotype) were transformed using the floral dip method [32]. Twenty transgenic lines of *GmARI1* were obtained. Eight T3 lines of the transgenic *GmARI1* Arabidopsis were examined by RT-PCR to select positive transgenic lines for further analyses.

### Al-tolerance of the *GmARI1* transgenic Arabidopsis

Homozygous T_3_ seeds of the transgenic lines and wild type plants were used for Al-tolerance analysis. Seeds were surfaced-sterilized as described before [33] and germinated on 1/2 MS medium for 7 days. The seedlings were transferred to 1/2 MS without Mg^2+^ but with 0 or 15 µM Al(NO_3_)_3_ and 8% Agar, pH 4.3, and then put on the medium with the plates placed vertically. After 15 days, the lengths of the roots were measured, and the Relative Root Growth (RRG) in each line was calculated as: RRG (%) =  (RL_Alt_- RL_Al0_)/(RL_ct_- RL_c0_), in which RL_Al0_ represent the root length before Al treatment, RL_Alt_ represent the root length after 15 days of Al treatment (15 µM Al(NO_3_)_3_, pH 4.3), RL_c0_ represent the root length before growth on the control medium, and RL_ct_ represent the root length after growth on the control medium (0 µM Al(NO_3_)_3_, pH 4.3). The statistical analysis of the experimental data was conducted by t-test with SPSS [34].

## Results

### Isolation and sequence analysis of *GmARI1* gene

The cDNA of *GmARI1* gene is 2043 bp in length, containing an open reading frame (ORF) of 1761 bp, a 112 bp 5′-untranslated region (UTR), and a 170 bp 3′-UTR. Its homologous gene, *GmARI2*, shares 97% of similarity in the nucleotide sequences of the ORF region. The *GmARI1* and *GmARI2* nucleotide sequence and the predicted amino acid sequence have been deposited in GenBank (accession number JX392390 and JX392391). The genomic sequence of *GmARI1* and *GmARI2* variants in cultivar Williams 82 have 15 exons and 14 introns (http://www.phytozome.net/soybean.php), which located on chromosome 11 and 12, respectively.

**Figure 1:**
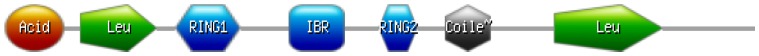
The protein structure of GmARI1. **Acid**: acid-rich cluster; **Leu**: Leu-rich cluster; **RING1**: a C3HC4 RING-finger; **IBR**: In Between RING fingers (IBR); **RING2**: a second C3HC4 RING finger; **Coile**∼: Coiled coil.

The deduced protein of *GmARI1* comprises 586 amino acids with the predicted molecular mass of 66.99 kDa and isoelectric point of 5.37. GmARI1 protein has a RBR domain, which contains an IBR (C6HC) domain flanked by RING1 and RING2 (C3HC4) (Fig. 1). The secondary structure of GmARI1 protein is predicted to be composed of 45.56% alpha helix, 11.09% extended strand, 41.64% random coil, and 1.71% beta turn.

**Figure 2:**
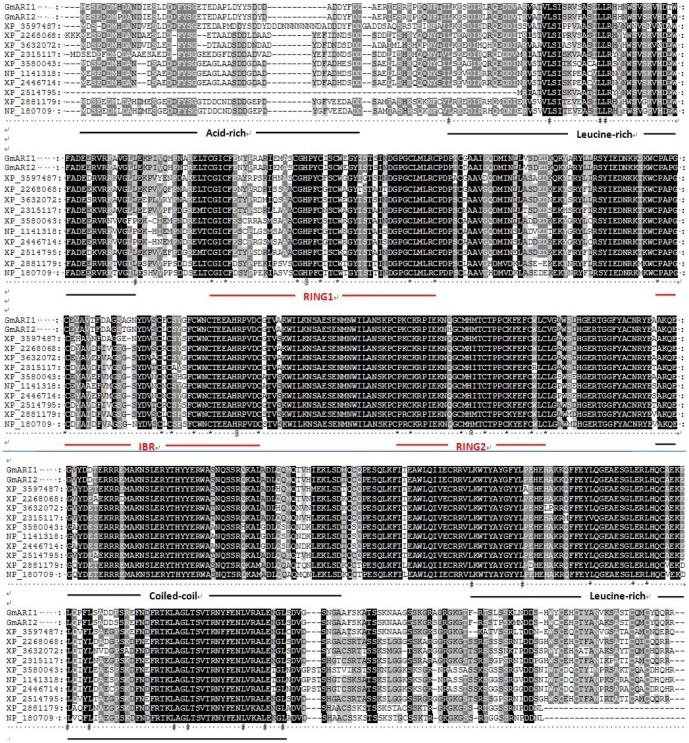
Multiple sequence alignment of GmARI1 with other RBR domain containing proteins. XP_3597487: *Medicago truncatula*; XP_2268068 and XP_3632072: *Vitis vinifera*; XP_2315117: *Populus trichocarp*; XP_3580043: *Brachypodium distachyon*; NP_1141318: *Zea mays*; XP_2446714: *Sorghum bicolor*; XP_2514795: *Ricinus communis*; XP_2881179: *Arabidopsis lyrata*; NP_180709: *Arabidopsis thaliana*; *: Cys; @: His; #: Leu and Ile.

**Figure 3:**
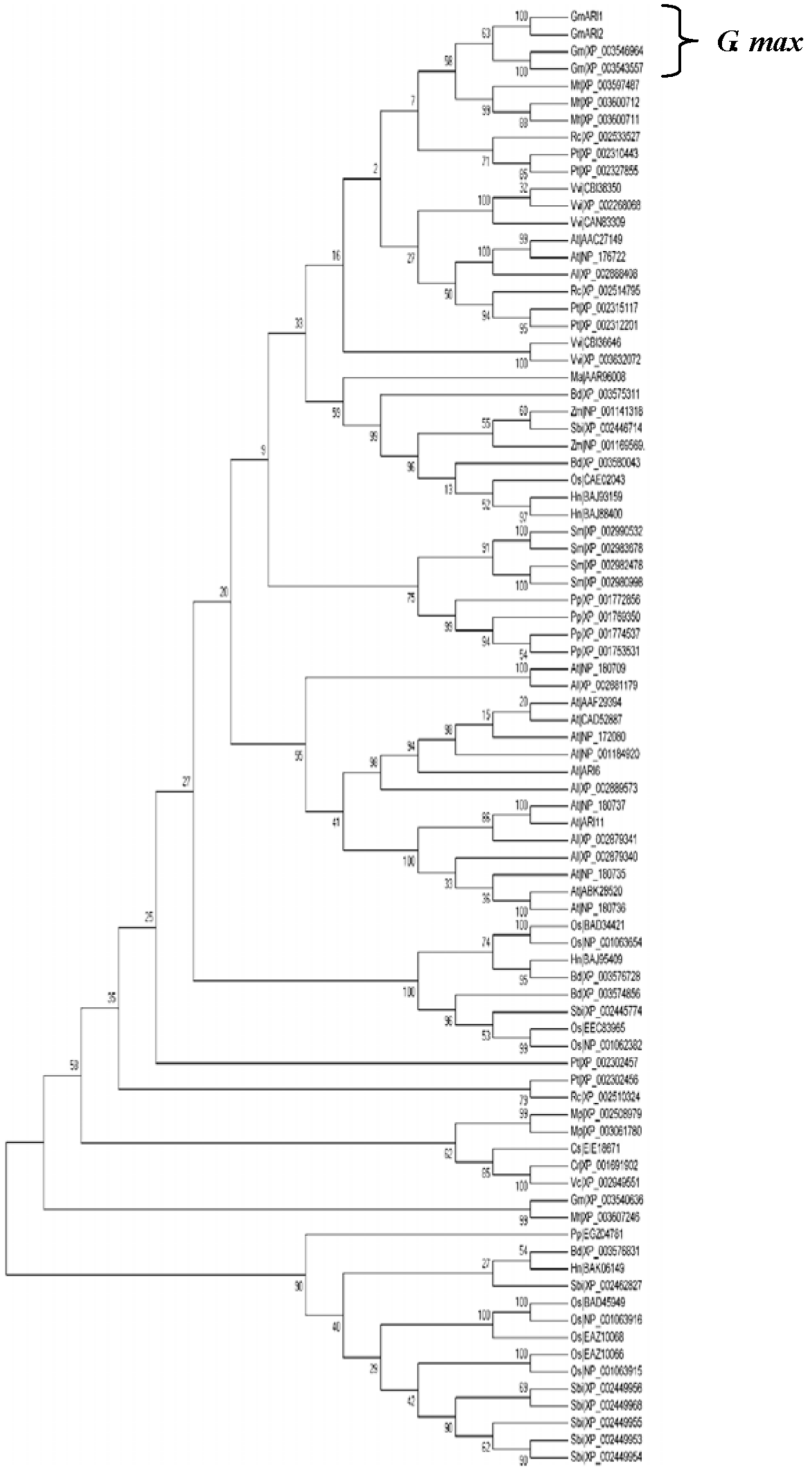
Phylogenetic relationships among soybean ARI proteins and other RBR domain containing proteins. The numbers on the tree indicate percent bootstrap values. The species abbreviations are listed as follows; *Al: Arabidopsis lyrata; At: Arabidopsis thaliana; Bd: Brachypodium distachyon; Cr: Chlamydomonas reinhardtii; Cs: Coccomyxa subellipsoidea; Hn: Hordeum vulgare; Ma: Musa acuminata; Mp: Micromonas pusilla; Mt: Medicago truncatula; Nt: Nicotana plumbaginifolia; Os: Oryza sativa subsp. Japonica; Pp: Physcomitrella patens; Pt: Populus trichocarpa; Rc: Ricinus communis; Sbi: Sorghum bicolor; Sm: Selaginella moellendorffii; Vc: Volvox carteri, Vvi: Vitis vinifera.*

In order to determine the relationship of GmARI1 and other RBR proteins, Blastp on Uniprot was used to search the homologues proteins of GmARI1. The top 87 amino acid sequences with RBR conserved domain were selected from different plant species, including *Arabidopsis thaliana*, *Medicago truncatula*, *Ricinus communis*, and *Zea mays L*. Multiple sequence alignment of these 87 amino acid sequences was performed. In addition to the ARI proteins from soybean which showed high similarity with *GmARI1*, the protein from *M. truncatula* showed 80% similarity with *GmARI1* (Fig. 2) The phylogenetic tree was drawn using MEGA 5.0 program based on Neighbor-Joining (NJ) with 1000 bootstrap replications (Fig. 3), which also showed ARI proteins from *G. max* was closely related to the protein from *M. truncatula*.

### Tissue expression pattern of *GmARI1* and the subcellular localization of its protein

**Figure 4:**
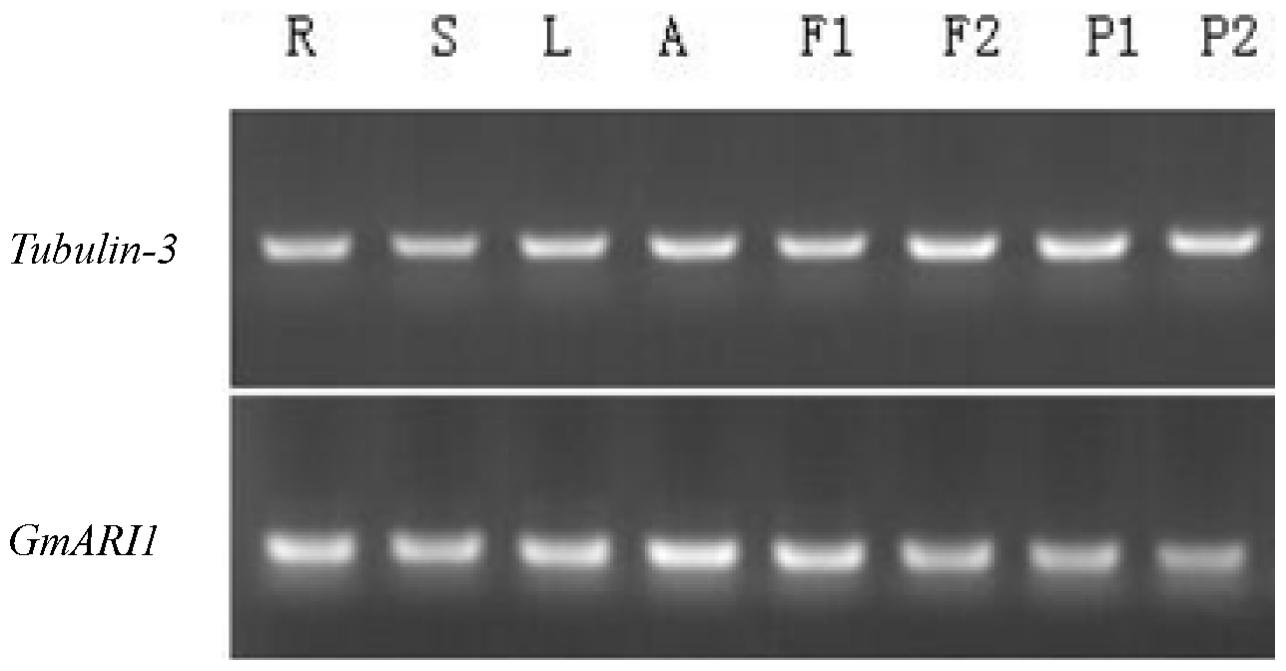
Tissue expression patterns of *GmARI1* in soybean. R: Roots; S: Stems; L: Leaves; A: Apex (SAMs); F1, F2: Flowers; P1, P2: Pods.

**Figure 5:**
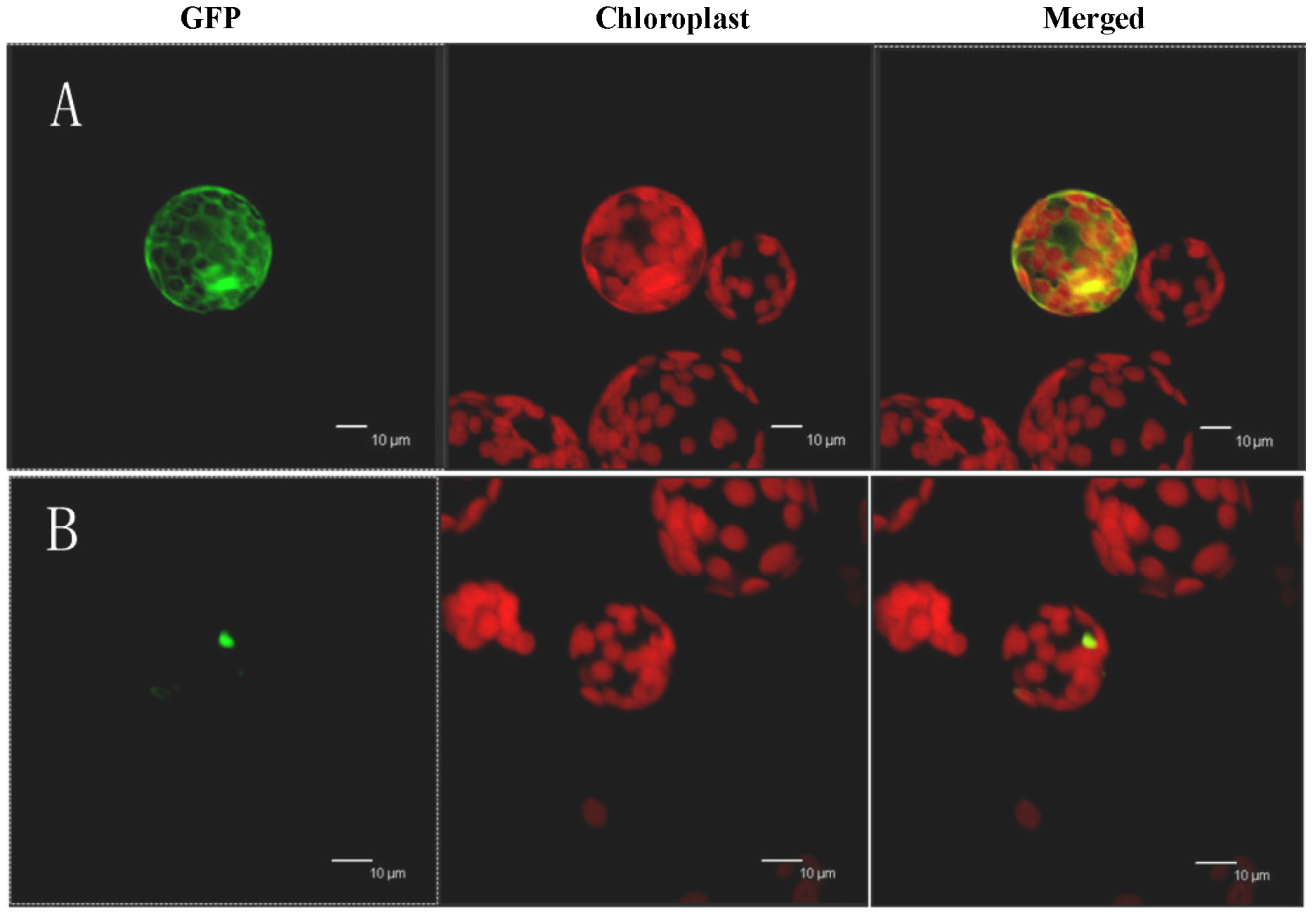
Sub-cellular localizations of GmARI1 protein. **A**: 35S-GFP; **B**: 35S-GmARI1-GFP.

*GmARI1* gene was expressed in roots, stems, leaves, SAMs, flowers, and pods (Fig. 4). We determined the subcellular localization of the GmARI1 protein by transient expression of *35S*-*GmARI1-GFP* in Arabidopsis protoplasts. GmARI1 was located in the nucleus, while the GFP control was mainly located in the cytoplasm (Fig. 5).

### Expression pattern of *GmARI1* under stress and plant hormone treatments

**Figure 6:**
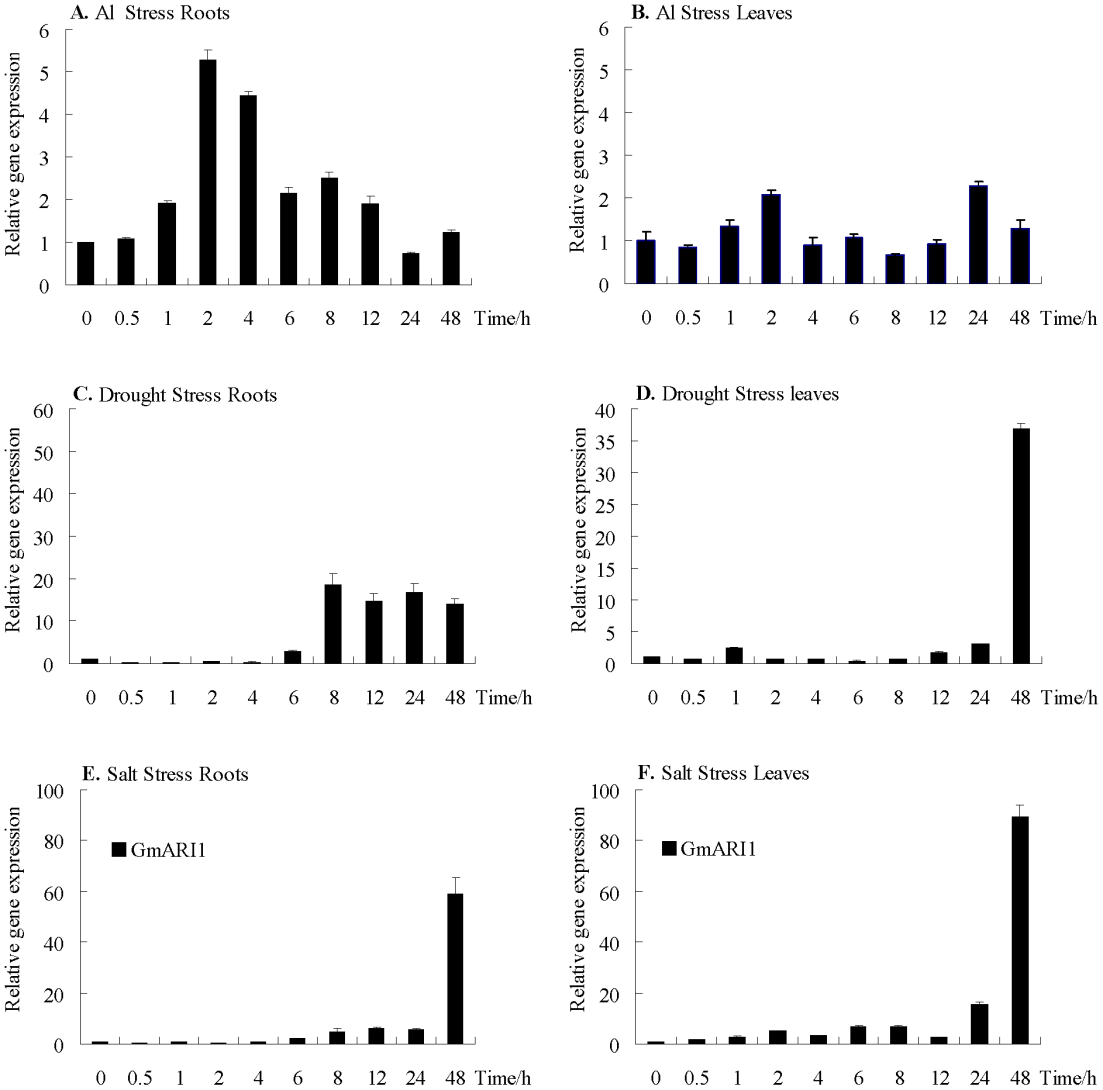
Relative gene expression levels of *GmARI1* in soybean under simulated stress treatments. **A**. relative gene expression in roots under Al stress (10 µM Al(NO_3_)_3_, pH4.3) **B**. relative gene expression in leaves under Al stress (10 µM Al(NO_3_)_3_, pH4.3) **C**. relative gene expression in roots under drought stress (400 mM mannitol) **D**. relative gene expression in leaves under drought stress (400 mM mannitol) **E**. relative gene expression in roots under salt stress (200 mM NaCl) **F**. relative gene expression in leaves under salt stress (200 mM NaCl). Error bars represent the standard error of three replicates.

**Figure 7:**
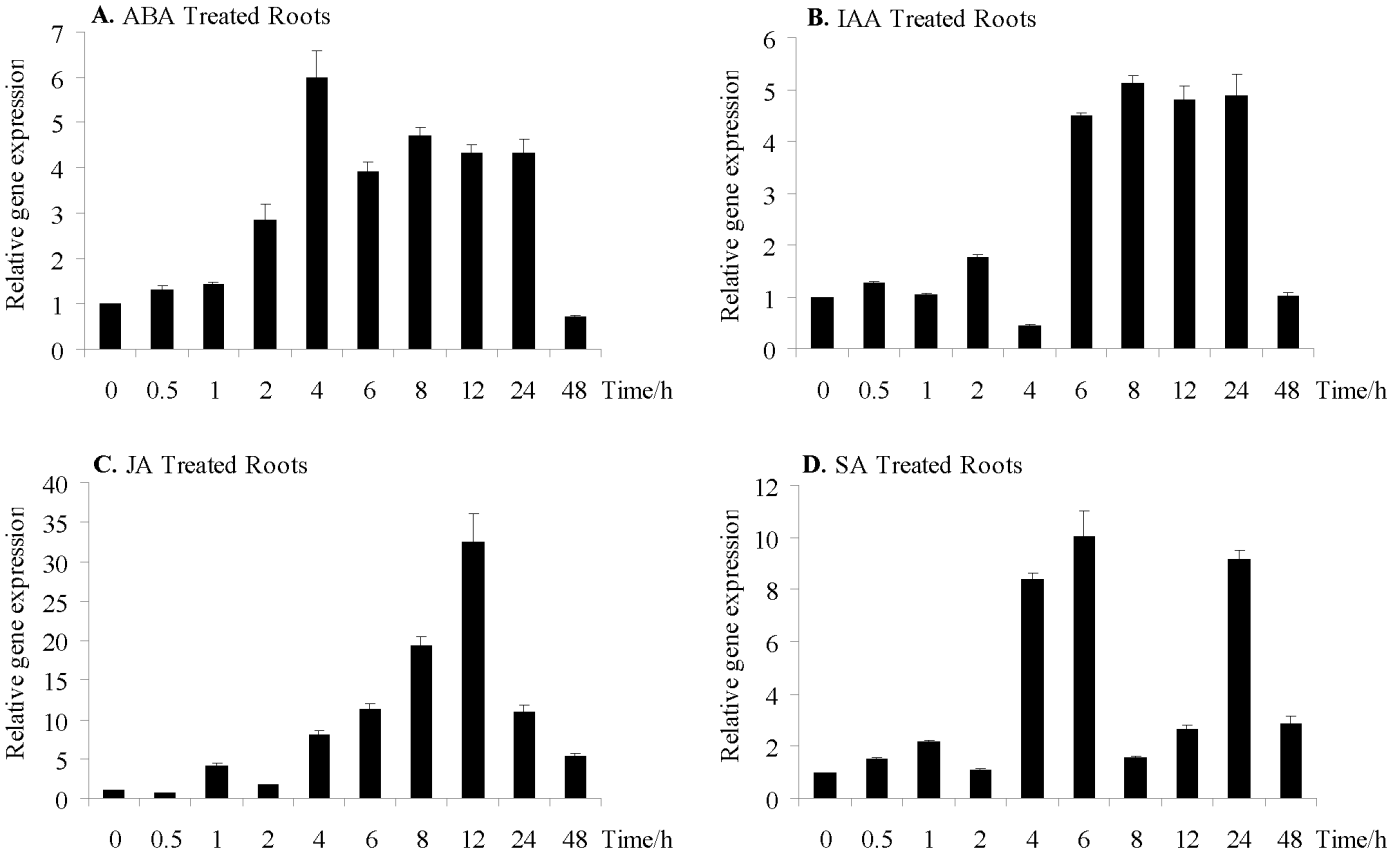
Relative gene expression levels of *GmARI1* in the roots of soybean after exogenous application of plant hormones. **A**. 100 µM ABA treatment **B**. 100 µM IAA treatment **C**. 100 µM JA treatment **D**. 150 µM SA treatment. Error bars represent the standard error of three replicates.

Real-time quantitative PCR was carried out to examine the expression pattern of *GmARI1* gene in soybean under different treatments including various abiotic stresses and plant hormones (Fig. 6 and 7). Under 10 µM Al (pH4.3) stress, the transcripts of *GmARI1* in soybean roots increased and peaked during 2 h to 4 h, but the transcripts in leaves did not change significantly (Fig. 6A, B). When treated with 400 mM mannitol (osmotic stress), the *GmARI1*gene expression was induced from 6 h to 48 h in roots, while showed delayed induction in leaves (Fig. 6C, D). Under 200 mM NaCl stress, the *GmARI1* gene expression showed a high induction after 24 h in both roots and leaves (Fig. 6E, F).

The transcriptional changes of *GmARI1* in response to ABA, IAA, JA and SA were also investigated in soybean roots. The transcripts of *GmARI1* were up-regulated during 2-24 h after ABA treatment (Fig. 7A). *GmARI1* was induced after 6 h IAA treatment and its transcript abundance was maintained at a higher level until 24 h (Fig.7B). The expression of *GmARI1* showed continual increase from 4 h and peaked around 12 h after JA treatment (Fig. 7C). Increased transcripts of *GmARI1* were detected at 4 h, 6 h and 24 h after SA treatment (Fig. 7D).

### E3 ubiquitin ligase activity of GmARI1 protein

**Figure 8:**
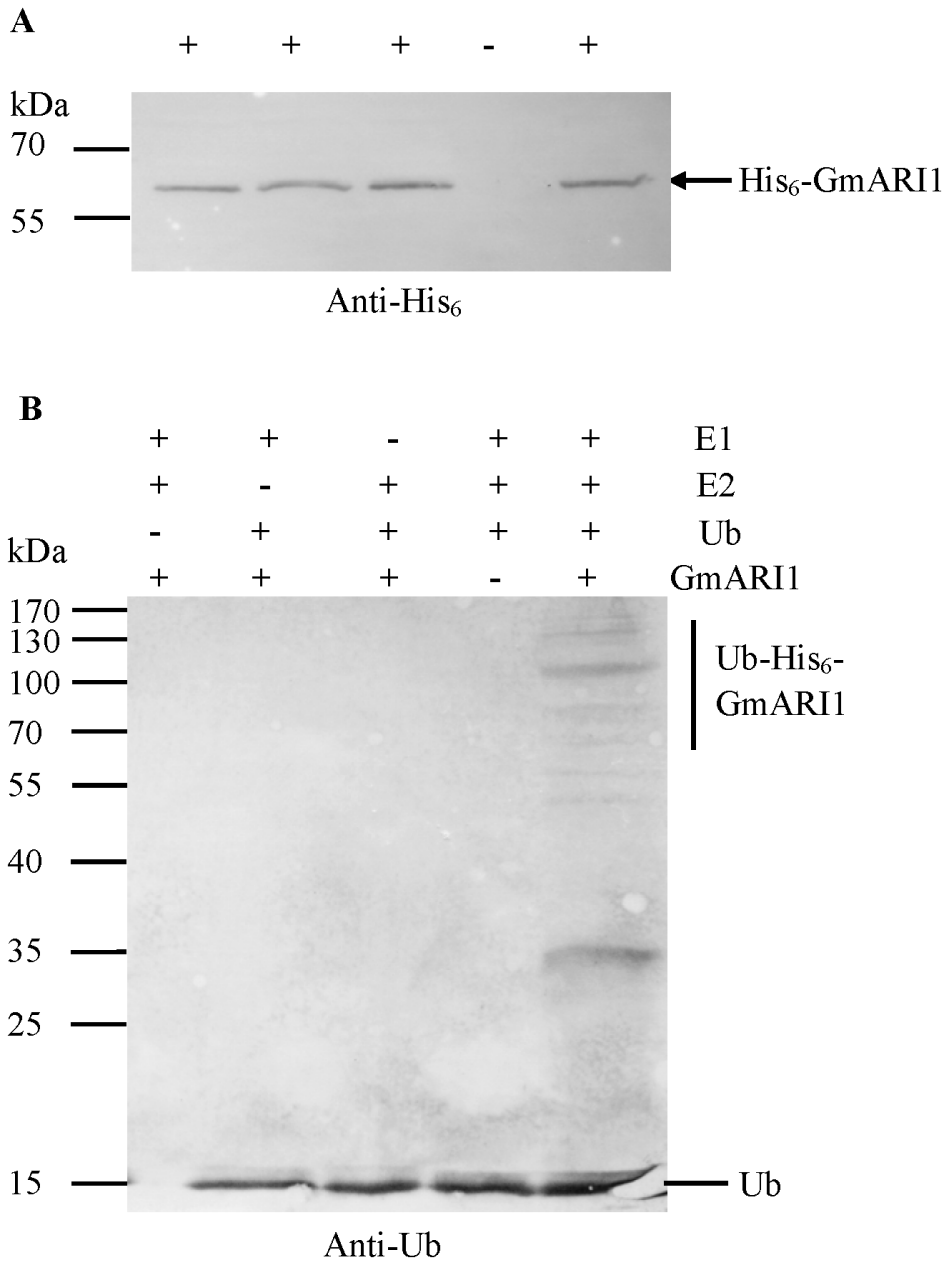
Western blot analysis of GmARI1 protein. **A**. Western blot using anti-His6 monoclonal antibody in the presence (+) or absence (−) of the purified His6 tag-GmARI1 proteins. **B**. In vitro E3 ubiquitin ligase activity assay of GmARI1 protein. His6 tag-GmARI1 fusion proteins were assayed for E3 activity in the presence of rabbit E1, human E2 (UbcH5b), and ubiquitin (Ub). The left numbers denote the molecular masses of marker proteins in kilodaltons.

To test if GmARI1 has the E3 ubiquitin ligase activity, a full-length GmARI1 protein with maltose binding protein (6× His tag) was expressed in *E. coli* and subsequently affinity-purified (His_6_-GmARI1) from the soluble fraction (Fig. S1). The purified recombinant His_6_-GmARI1 protein was about 66 kDa as expected, and the western blotting with anti-His_6_ monoclonal antibody also showed the purified target recombinant protein had the right molecular weight of 66 kDa (Fig. 8A). In vitro self-ubiquitination assays were performed in the presence of rabbit E1, human E2 (UbcH5b), and Ub (Fig. 8B). Polyubiquitination was detected only in the presence of E1, E2, Ub and His_6_-GmARI1. A negative result was observed if either E1, E2, Ub or His_6_-GmARI1 was omitted in the reaction. These results indicate that GmARI1 has E3 ubiquitin ligase activity.

### Performance of transgenic plants over-expressing *GmARI1* under Al stress

**Figure 9:**
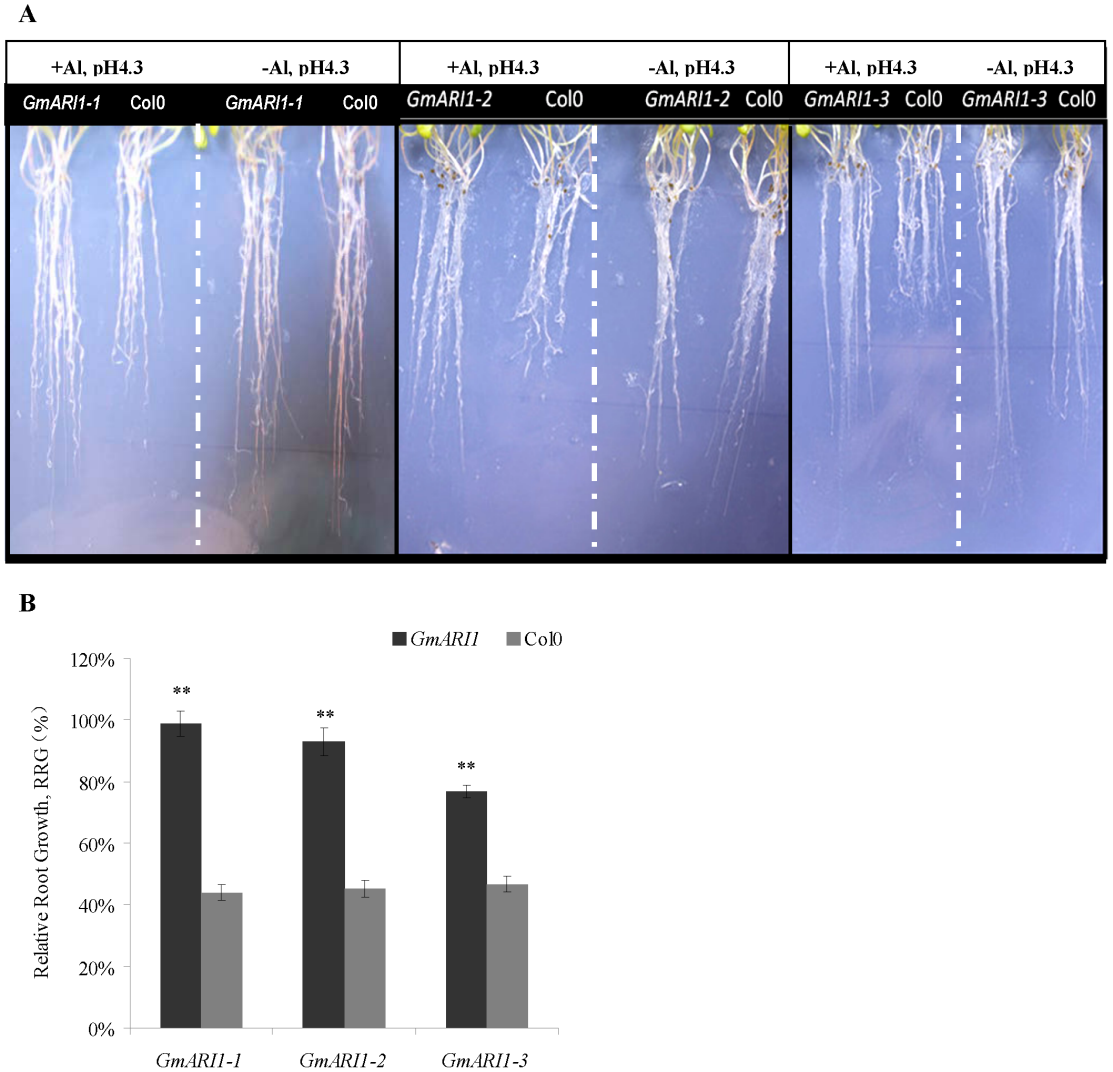
Performance of the transgenic Arabidopsis under 15 µM Al treatment. **A**. Root phenotypes of the *35S: GmARI1* overexpression lines *GmARI1-3* and the wild type Col-0 grown on 15 µM Al in 1/2 MS (+Al, pH4.3), and 0 µM Al in 1/2 MS (-Al, pH4.3). **B**. Relative root growth (RRG, %) of the transgenic Arabidopsis lines (*GmARI1-1, GmARI1-2, GmARI1-3*) and the wild type (Col-0). RRG was calculated by the root growth length under Al treatment (15 µM Al in 1/2 MS, pH4.3) divided by the root growth length under control (0 µM Al in 1/2 MS, pH4.3). Error bars represent the standard error (SE), ** indicate the significance level of 0.01 by t-tests.

The gene expression of *GmARI1* was induced early by Al stress, therefore we further studied its function in transgenic plants. An expression plasmid vector of *pMDC83-GmARI1* was constructed and introduced into Arabidopsis plants using floral dip method. Transgenic T_3_ Arabidopsis over-expressing *GmARI1* were generated and the positive transgenic lines were identified by RT-PCR (Fig. S2). Seeds of three T_3_ homozygous transgenic lines (*GmARI1-1, GmARI1-2*, and *GmARI1-3*) and wild type Col-0 were germinated on 1/2 MS medium. After 10 days, the seedlings were transferred to 1/2 MS (pH4.3) containing 15 µM Al or 0 µM Al as a control. Fifteen days later, the root growth of the wild type Col-0 was severely inhibited by 15 µM Al as compared with the control medium (0 µM), while the transgenic lines were little affected by Al (Fig. 9 A). The relative root growth (RRG) of the transgenic lines was significantly (*p*<0.01) longer than the wild type under Al treatment (Fig. 9B). The relative abundance of *GmARI1* in the transgenic lines of *GmARI1-1* and *GmARI1-2* is higher than *GmARI1-3*, which coincided with RRG result (Fig. S3 and Fig. 9B).

## Discussion

RING-type E3 ubiquitin ligases play important roles in plant responses to abiotic stresses [7], [8], [35]. RBR subclass of RING-containing E3 ligases were recently shown as an important group of proteins since the discovery of *parkin*, a mutation causing the familial autosomal-recessive juvenile parkinsonism (AR-JP) [36]. However, there is little research on the plant RBR family. The RBR family was classified into 14 subfamilies, including Ariadne (ARI), ARA54, Dorfin, parkin, PlantI, PlantII, and XAP3 [9], but the function of ARI class is largely unknown. In this study, *GmARI1* gene was isolated and characterized from soybean. Based on the analysis of predicted protein domains, *GmARI1* gene belongs to the Ariadne subfamily, characterized by the presence of an N-terminal acid-rich cluster, followed by a RBR domain and a Coiled coil region at the C-terminus [17]. The origin of Ariadne can be traced back earlier in time, as it was found not only in animal, fungal, and plant, but also in some protist [37]. The blastp result indicated among the RBR domain containing protein from other species, *Medicago truncatula* had the highest similarity (80%) with *GmARI1* and other ARI proteins from soybean.

*GmARI1* gene was expressed ubiquitously in roots, stems, SAMs, leaves, flowers, and pods of soybean plants. The GmARI1 protein was located in the nucleus of the cell. Real-time quantitative PCR showed that the expression level of *GmARI1* in soybean root under Al (10 µM, pH 4.3) treatment reached the peak during 2 to 4 hours, but no significant change was detected in the leaves. This coincides with the fact that Al toxicity was first and mainly acting on plant roots [38], limiting water and nutrition absorption [39], which further inhibiting the development of whole plant and reducing yield [40], [41]. Therefore in the early stage (less than 4 hours) of Al stress, induced gene expression of *GmARI1* in soybean roots may play important roles to trigger downstream signaling pathways to protect root cells from Al toxicity.

It has been reported that Al induces oxidative stress and DNA damage in plant cells [42]–[44]. Reactive oxygen species (ROS) constantly attack DNA, leading to oxidative DNA damage [45]. The cell cycle checkpoint regulators could detect and respond to such damage, leading to inhibition of root growth [46]. Protein ubiquitination is involved in DNA transcription and repair, cell cycle and division [1], and is emerging as a critical regulatory mechanism of DNA damage response [47]. Several RING domain-containing E3 ubiquitin ligases play an essential role in response to DNA damage [48] ^.^ In vitro ubiquitination assay showed GmARI1 has E3 ligase activity (Fig. 8), therefore we hypothesize that GmARI might be involved in the oxidative DNA damage repair to confer Al tolerance. We investigated the co-expression pattern of *GmARI* (http://bioinformatics.cau.edu.cn/SFGD/), and found Glyma02g15070 was on the top list of coexpression genes with *GmARI*. The homolog gene of Glyma02g15070 in arabidopsis is AT1G49670, which was shown to be involved in oxidative stress tolerance (http://www.arabidopsis.org/). Suppression of oxidative stress might help plants reduce the damage or root growth inhibition [49], [50]. Therefore, the Al tolerance observed in *GmARI* overexpression lines might be due to the improved tolerance to oxidative stress, or/and other signaling cascades.

The activities of cell wall-bound peroxidases in the annual legume, *Cassia tora*, significantly increased with Al concentrations, and were regulated by JA [51]. Another study showed that the SA-signaling and SA-dependent expression of a respiratory burst oxidase homolog gene is involved in Al responsive oxidative burst in Arabidopsis [52]. Here in this study, the expression of *GmARI1* was induced by Al stress, as well as JA and SA treatments in soybean roots. These suggested *GmARI1* might mediate soybean response to Al through oxidative species signals, which may overlap with plant hormone signaling pathways. The T_3_ transgenic Arabidopsis plants over-expressing the *GmARI1* gene showed significant improvement in Al tolerance comparing with wild type plants, which further support the important role of *GmARI1* gene in plant response to Al stress.

## Supporting Information

Figure S1 **Expression and purification of the recombinant GmARI1 proteins.** The recombinant His6-GmARI1 proteins were expressed in *E.coli* BL21 (DE3) and analyzed by SDS–PAGE. Lane 1, total proteins from *E. coli* cells before IPTG induction; lane 2, total proteins containing pET28a-GmARI1 from *E. coli* cells after induction by IPTG; lane 3, purified recombinant His6-GmARI1 protein.
(DOC)

Figure S2 **RT-PCR confirmation of the transgenic Arabidopsis T3 lines GmARI 1 to 8.** (−): Arabidopsis wild ecotype Col-0; (+): plasmid pMDC83-GmARI1.
(DOC)

Figure S3 **Expression of the** ***GmARI1*** **gene in 2-week-old Arabidopsis plants quantified by qRT-PCR using actin (** ***AtACT2*** **) as the reference gene.** The Arabidopsis plants were germinated and grown on 1/2 MS medium (pH5.8) for two weeks and then transferred to 1/2 MS medium with 25 µM AlCl_3_ (pH4.3). Two hours later, tissues were sampled from the wild type Arabidopsis Col0 and homozygous transgenic lines separately (each sample was the mixture of four plants). Error bars are the standard errors from three replications.
(DOC)
