# Supplementary material for: Overexpression of a Soybean Ariadne-Like Ubiquitin Ligase Gene GmARI1 Enhances Aluminum Tolerance in Arabidopsis
Source: PLoS One. 2014 Nov 3;9(11):e111120. doi: 10.1371/journal.pone.0111120 (PMC4218711; doi:10.1371/journal.pone.0111120)
Supplement: Figure S1 — Expression and purification of the recombinant GmARI1 proteins. The recombinant His6-GmARI1 proteins were expressed in E.coli BL21 (DE3) and analyzed by SDS–PAGE. Lane 1, total proteins from E. coli cells before IPTG induction; lane 2, total proteins containing pET28a-GmARI1 from E. coli cells after induction by IPTG; lane 3, purified recombinant His6-GmARI1 protein. (DOC) [file pone.0111120.s001.doc]

35.8

97.6

66.4

44.3

**kDa**

Recombinant

His_6_-GmARI1 protein


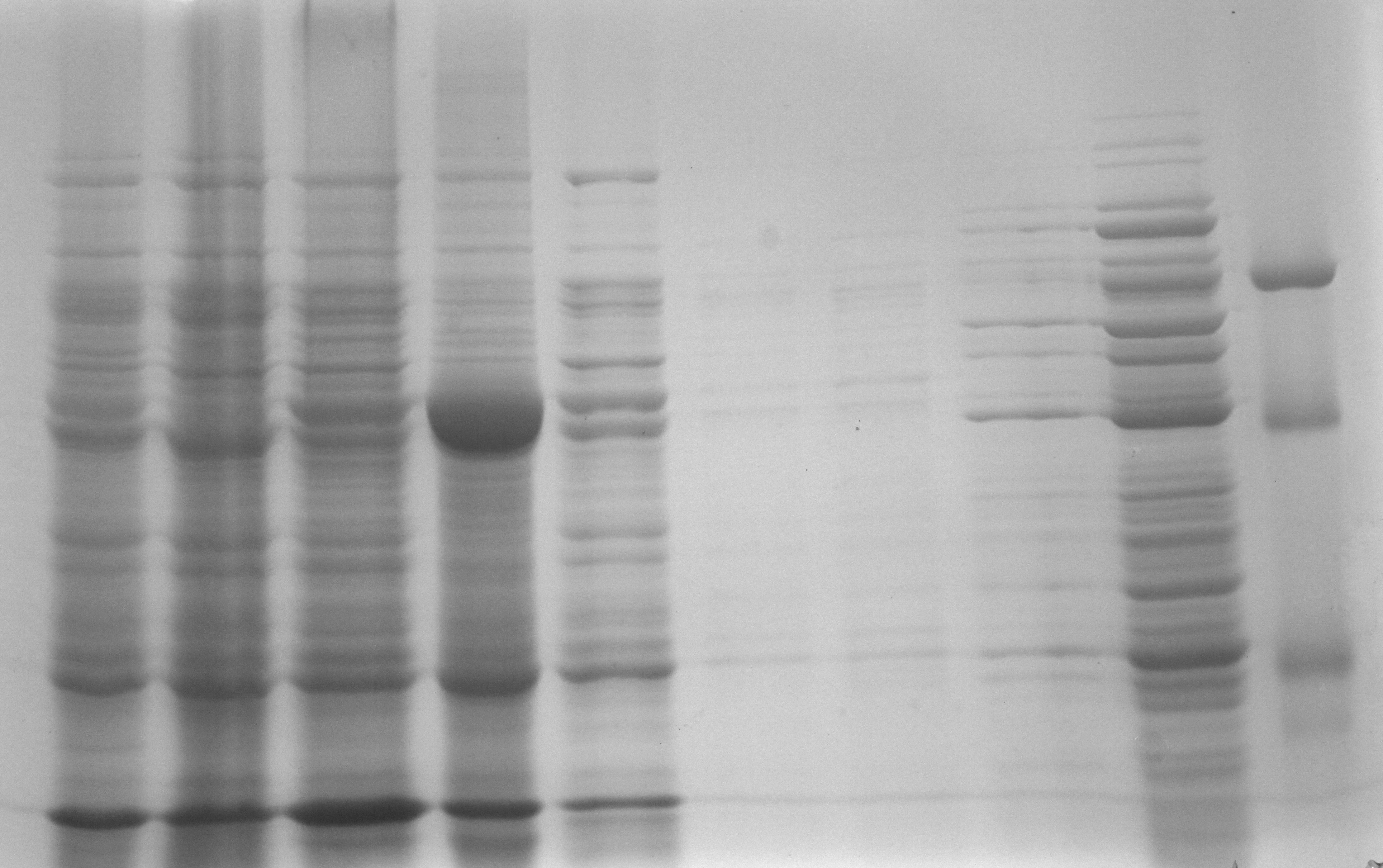


**Figure S1 Expression and purification of the recombinant GmARI1 protein.** The recombinant His6-GmARI1 protein was expressed in *E.coli* BL21 (DE3) and analyzed by SDS–PAGE. Lane 1, total proteins from *E. coli* cells before IPTG induction; lane 2, total proteins containing pET28a-GmARI1 from *E. coli* cells after induction by IPTG; lane 3, purified recombinant His6-GmARI1 protein.
