# Supplementary material for: Overexpression of a Soybean Ariadne-Like Ubiquitin Ligase Gene GmARI1 Enhances Aluminum Tolerance in Arabidopsis
Source: PLoS One. 2014 Nov 3;9(11):e111120. doi: 10.1371/journal.pone.0111120 (PMC4218711; doi:10.1371/journal.pone.0111120)
Supplement: Figure S2 — RT-PCR confirmation of the transgenic Arabidopsis T3 lines GmARI 1 to 8. (−): Arabidopsis wild ecotype Col-0; (+): plasmid pMDC83-GmARI1. (DOC) [file pone.0111120.s002.doc]

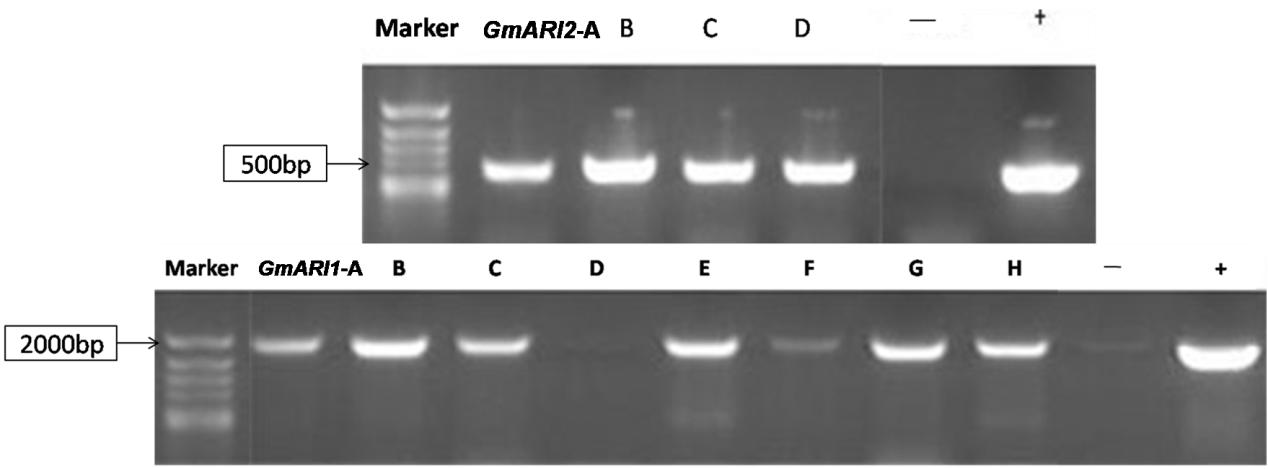


Marker 1 2 3 4 5 6 7 8 (-) (+)

Figure S2 RT-PCR confirmation of the transgenic Arabidopsis T3 lines GmARI 1 to 8. (-): Arabidopsis wild ecotype Col-0; (+) : plasmid pMDC83-GmARI1.
