# Supplementary material for: Overexpression of a Soybean Ariadne-Like Ubiquitin Ligase Gene GmARI1 Enhances Aluminum Tolerance in Arabidopsis
Source: PLoS One. 2014 Nov 3;9(11):e111120. doi: 10.1371/journal.pone.0111120 (PMC4218711; doi:10.1371/journal.pone.0111120)
Supplement: Figure S3 — Expression of the GmARI1 gene in 2-week-old Arabidopsis plants quantified by qRT-PCR using actin ( AtACT2 ) as the reference gene. The Arabidopsis plants were germinated and grown on 1/2 MS medium (pH5.8) for two weeks and then transferred to 1/2 MS medium with 25 µM AlCl3 (pH4.3). Two hours later, tissues were sampled from the wild type Arabidopsis Col0 and homozygous transgenic lines separately (each sample was the mixture of four plants). Error bars are the standard errors from three replications. (DOC) [file pone.0111120.s003.doc]

**Figure S3 Expression of the *GmARI1* gene in 2-week-old Arabidopsis plants quantified by qRT-PCR using actin (*AtACT2*) as the reference gene.** The Arabidopsis plants were germinated and grown on 1/2 MS medium (pH5.8) for two weeks and then transferred to 1/2 MS medium with 25 μM AlCl_3_ (pH4.3). Two hours later, tissues were sampled from the wild type Arabidopsis Col0 and homozygous transgenic lines separately (each sample was the mixture of four plants). Error bars are the standard errors from three replications.

Real-time qPCR was performed to analyze the relative gene expression levels of *GmARI1* in different transgenic Arabidopsis lines and wild type plants. Total RNAs were extracted from 2-week-old Arabidopsis plants using RNAprep pure Plant Kit (TianGen Biotech Co. Ltd.). The cDNA was synthesized using using PrimeScript RT (Perfect Real Time) Kit (TaKaRa), followed by real-time qPCR using SYBR Premix Ex Taq (TaKaRa) on a Roche LightCycler480 Real-Time PCP system. The relative abundance of GmARI1 in Arabidopsis plants was normalized with the Arabidopsis actin gene *AtACT2* (AT3G18780, Forward Primer: GCTGACCGTATGAGCAAAGA, Reverse Primer: GATCCACATCTGTTGGAACG) using the ΔCt method (Czechowski *et al*., 2005 and Ding *et al*., 2013).

Czechowski T., Stitt M., Altmann T., Udvardi M.K. and Scheible W.R. (2005) Genome-wide identification and testing of superior reference genes for transcript normalization in Arabidopsis. Plant Physiol. 139, 5–17.

Ding ZJ., Yan JY, Xu XY, Li GX and Zheng SJ (2013) WRKY46 functions as a transcriptional repressor of ALMT1, regulating aluminum-induced malate secretion in Arabidopsis. The Plant J. 76: 825–835.
